# Interpretable Ensemble Architectures with Theory-Informed Features for High-Fidelity Real-Time Congestion Forecasting on the Chalong Rat Expressway

**DOI:** 10.3390/s25227090

**Published:** 2025-11-20

**Authors:** Pongphatana Puttima, Tongtong Zhou, Zhihua Chen

**Affiliations:** 1 School of Ocean and Civil Engineering, Shanghai Jiao Tong University, 800, Dongchuan Road, Minhang District, Shanghai 200240, China; winpong11@sjtu.edu.cn; 2 School of Design, Shanghai Jiao Tong University, 800, Dongchuan Road, Minhang District, Shanghai 200240, China; tongtz@sjtu.edu.cn

**Keywords:** real-time congestion forecasting, theory-guided feature engineering, cell transmission model, Kerner’s three-phase traffic theory, Helbing microscopic dynamics, hybrid random forest–XGBoost, SHAP analysis

## Abstract

Accurately forecasting traffic congestion on urban expressways remains challenging, especially under unstable flow conditions where conventional machine learning models often suffer from reduced accuracy and interpretability. This study introduces a domain-theoretic machine learning framework designed for real-time congestion prediction on the Chalong Rat Expressway in Bangkok, Thailand. Feature engineering incorporates principles from the macroscopic cell transmission model, Kerner’s three-phase theory, and Helbing’s microscopic dynamics to capture key interactions such as density–flow relationships, jam propagation, and driver response gradients. A hybrid random forest–XGBoost ensemble is developed and evaluated against standard machine learning baselines. The results demonstrate that the proposed ensemble achieved superior performance across mean absolute error (MAE), root mean square error (RMSE), coefficient of determination (R^2^), and prediction interval coverage (PICP), particularly near congestion transition boundaries. SHapley Additive exPlanations (SHAP) analysis confirmed corrected outflow, jam speed, and repulsive force as dominant predictors, underscoring the model’s interpretability. By integrating traffic theory with interpretable machine learning, this framework enables accurate, explainable, and deployable real-time congestion forecasting for intelligent transportation systems.

## 1. Introduction

**Figure 1:**
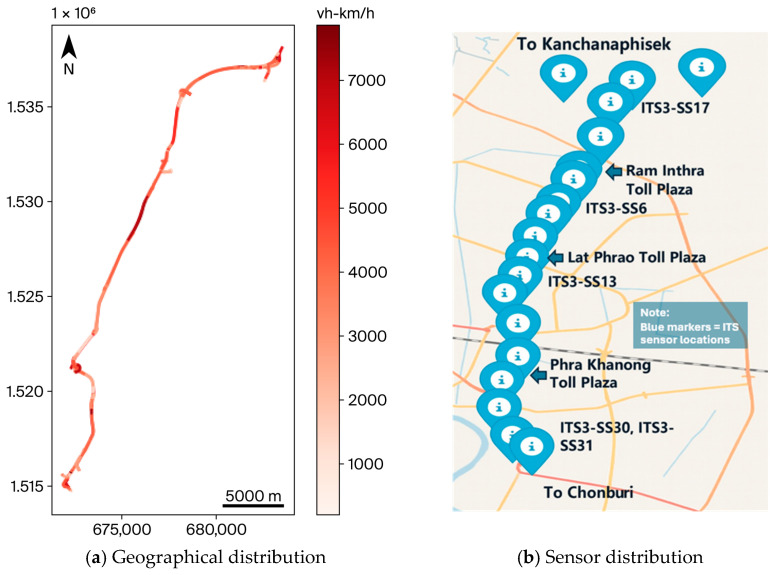
Locations of ITS infrastructure along the Chalong Rat Expressway: (**a**) The spatial distribution of ITS sensors along the corridor; (**b**) the geometrical alignment of the Chalong Rat Expressway.

Urban expressways offer an immensely high-speed setting for vital corridors expected to decongest ground-level roads. On this front, the Chalong Rat Expressway runs for 28.2 km, acting as a connector for north-eastern arterial roads in Bangkok. The Expressway Authority of Thailand (EXAT) installed microwave detectors on this expressway for traffic monitoring purposes [1]. Intelligent transportation system (ITS) sensors are used to capture the real-time spatiotemporal traffic dynamics distributed spatially along the Chalong Rat corridor, as shown in Figure 1.

The Chalong Rat Expressway acts as a principal urban corridor that lies amidst Bangkok city and is fitted with traffic management ITS sensors that record flow, speed, and density [1]. Numerous studies have analysed expressway conditions with the help of sensor data. Leunpech et al. examined the effect of a proposed speed limit on urban expressways, using secondary data collected from 33 microwave detectors that aggregated flow, speed, and density information in one-minute intervals [2]. Another study used simulation data generated using AIMSUN and artificial neural networks to create an incident detection network for Chalong Rat, with density, speed, occupancy, flow, and headway as tunable parameters [3]. These studies showed that high-resolution ITS data exist and that ML can be applied in this corridor. However, these studies were geared toward simulation or policy evaluation rather than dynamic real-time congestion forecasting. At a more general level, machine learning and ensemble techniques have been widely used for traffic forecasting. An interpretable ensemble-tree-based traffic flow forecasting paradigm has been proposed, claiming to hold both accuracy and explainability [4]. A stacking-based learning architecture has been introduced to maintain the balance between prediction accuracy and transparency [5]. An ensemble deep learning model has been suggested for long-term traffic flow prediction, highlighting robustness benefits [6]. Hybrid methods such as those introducing autoregressive terms into ensemble models, have also been shown to perform well with respect to highway traffic datasets [7]. However, studies on expressway forecasting of socioeconomic importance to Bangkok are scarce [2,3]. Most high-performance models are black-boxes that possess minimal interpretability, thus impeding their operational adoption [4,5,6]. To the best of the authors’ knowledge, uncertainty quantification is practically never used in forecasting models, although it is a key factor in decision-making in dynamic traffic environments [7]. Robustness over traffic regimes, such as peak and off-peak times or high- and low-congestion instances, is under-explored, with model performances usually being subject to variation under such circumstances.

Datasets have previously been utilized to evaluate speed limit policies and for incident detection based on simulated data. However, congestion forecasting in real time with data from operational detectors is still an emerging technique. Many advanced ML and DL algorithms are highly accurate, but they are black-boxes from an operational decision-making standpoint [4,5,6]. Uncertainty quantification is seldom included, so operators remain devoid of calibrated confidence bounds important for risk-aware control [7,8]. Even though several simulation-based predictions have been made for Bangkok’s expressway, such as Aimsun Live [9], an interpretable, uncertainty-aware, detector-driving forecasting method oriented toward the Chalong Rat Expressway has not yet been put forward.

To bridge such research gaps, the present study provides interpretable ensemble architectures with an embedded theory-informed feature selection that can ensure greater fidelity and real-time forecasts of congestion on the Chalong Rat Expressway. Using an ITS sensor, ensemble learning, and conformal prediction for uncertainties, the study tries to predict congestion with accuracy and uncertainty while endorsing the most important features of traffic. The main contributions of the study are as follows:

- It proposes theory-guided feature engineering in which CTM, three-phase, and Helbing dynamics are combined with raw expressway data.
- It proposes the hybrid RF–XGBoost method with autoregressive embedding (“Hybrid-AR” and “Final Blend”), which outperforms the standard ML baselines.
- Robustness is tested with respect to peak/off-peak and congestion regimes, with statistical significance confirmed via bootstrap.
- Conformal prediction is applied for calibrated uncertainty intervals suitable for ITS deployment.
- It provides explainability using SHAP, and the results are consistent with traffic flow theory.
- It delivers a replicable end-to-end workflow from raw data preprocessing to real-time forecasting and visualization.

## 2. Related Work

### 2.1. Interpretable and Theory-Informed Models

Interpretability is one of the most critical research aspects in the application of traffic forecasting in real-time operations. Tree-ensemble forecasting with clear feature attribution demonstrates interpretable ensembles that can achieve accuracies comparable to deep learning while maintaining explainability for an ITS deployment [4]. Stacking-based ensembles with multiple weak learners improve prediction accuracy while providing transparent contributions from each model for urban traffic forecasting [5]. Deep ensemble approaches for long-term traffic flow prediction show that ensemble averaging reduces variance and enhances robustness in extended horizon forecasts [6]. ARIMA and random forests couple the autoregressive structure of the system with nonlinear feature manipulation when predicting flows on highways [7]. Multi-regime ensemble learning dynamically adapts its model according to traffic regimes and thus significantly reduces prediction errors in complex networks [10]. These methods provide multimodal explainable prediction models to bridge various inputs, such as traffic counts, weather, and events, with interpretable outputs to improve transparency in operations [11]. Between signal decomposition, functional PCA, and ensemble integration, one can clean the signals and improve the forecast to obtain interpretable patterns at various temporal resolutions [12]. These works jointly imply that ensemble architectures coupled with traffic theory or signal decomposition techniques can yield performance-oriented and interpretable forecasting systems ready for deployment.

Although recent deep learning models have offered even better forecasting performance, they have frequently compromised trainability to accommodate for their complexity. Wei et al. [13] developed the STGSA model which utilizes graph attention and multi-path aggregation to combine spatial and temporal dependencies, and improved accuracy on large scale networks to demonstrate it. Qian et al. [14] introduced the multi-task iTransformer model which utilized saturation-based modelling and event-induced variability in available capacity based on the task, in order to produce better short-term congestion predictions. Liu et al. [15] used a Dandelion Algorithm as part of a community-based feature selection method, employed as part of a broad learning system (BLS) to give a lightweight and interpretable deep learning combined with ensemble approaches for predicting traffic flow. These three methods are all strong predictive performance approaches; however, due to their complicated architecture or custom specificity, they do not provide model transparency to support implementation in a practical real-time ITS context. The framework proposed in this work is intended to facilitate model interpretability through theory-informed features, ensemble blending and calibrated uncertainty, while still realizing a substantive determinate of real-time accuracy and overall deployed readiness progress.

### 2.2. Uncertainty-Aware Forecasting

Uncertainty quantification (UQ) is required to implement traffic management operations successfully. Online adaptive short-term flow estimation frameworks create random uncertainty intervals that respond flexibly to time-varying conditions [7]. Attention-based hybrid deep networks, together with Bayesian inference, produce probabilistic forecasts with credible intervals for real-time ITS control [8]. Deep ensemble GNNs quantify epistemic and aleatoric uncertainties in transitioning states, and they are quite unpredictable when applied to freeways [16]. Spatiotemporal GNN ensembles produce better calibration and robust uncertainty quantification on large highway networks [17]. An adaptive modelling framework considers non-stationary traffic dynamics to provide resilience in a dynamic environment [18]. Conformal prediction yields distribution-free guarantees on prediction intervals, allowing for robust intervals for real-world highway forecasts [19]. Relational conformal prediction methods further strengthen the calibration of uncertainty by considering subsisting correlation structures among traffic time series [20]. Together, the above techniques present a clear transition from deterministic frameworks to probabilistic and risk-aware ones, underscoring UQ as the key link between prediction models and operational decision-making.

### 2.3. Expressway and Bangkok Corridor Studies

Bangkok expressway studies demonstrate the potential and shortcomings of different strategies. Incident detection schemes based on simulation have been carried out on Chalong Rat with artificial neural networks trained on simulated data as opposed to real-time actual observations [3]. Support vector machine and data mining-based traffic prediction models for Bangkok have reached a congestion classification accuracy above 90%, demonstrating the feasibility of local ML approaches [21]. Spatial–temporal patterns of congestion revealed by traffic state analysis of Bangkok expressways are instructive for forecasting design [22]. Studies on the time headway distribution of Bangkok expressways have provided insights into local driver behaviours affecting the modelling of the fundamental diagram [23]. A multi-sensor dataset from the Bangkok CBD intersection combining loops, CCTV, and thermal sensors has served to analyse inbound–outbound traffic, thereby enriching the scope of local data availability [24]. The anomaly detection applied to the Chalerm Mahanakhon Expressway has substantiated the need for web-based monitoring and brought to light recurring problems with data reliability [25]. Studies of loop detector validation and imputation have demonstrated that preprocessing methods need to be more robust under expressway conditions [26]. A review of temporal imputation techniques found that advanced imputation is better than naive filling in preserving traffic dynamics [27]. Collectively, Bangkok is a city endowed with data richness and institutional readiness [9], but a more interpretable, uncertainty-aware, detector-driven forecasting framework tailor to the Chalong Rat Expressway is still needed.

### 2.4. Data Quality and ITS Reliability

The quality and reliability of sensor data are leading factors in determining the performance of a traffic forecasting model. Expressway loop detectors may suffer from problems such as missing values, malfunctions, and erroneous readings, all of which may lead to biased predictions regarding accuracy and stability if not addressed [26]. A systematic review of imputation techniques has demonstrated that more advanced methods like matrix factorization and deep learning imputation will outperform simple interpolation in maintaining spatiotemporal dynamics [27]. Anomaly detection research in Bangkok corroborates the fact that reliability issues remain in web-based traffic state data, thus highlighting the necessity for robust preprocessing pipelines [25]. Combining signal decomposition with functional PCA shows that denoising improves not only prediction accuracy but also interpretability under congested regimes [12]. Thus, a robust validation scheme and anomaly detection and imputation methods will need to form the core building blocks for a reliable and interpretable framework for forecasting expressway operations.

Prior to this major advancement in traffic forecasting research, most studies pertaining to the Bangkok expressway network, including the Chalong Rat Expressway, focused more on simulation analyses or policy evaluations and less on operational, data-based forecasting. Existing machine learning and deep learning models have reasonable predictive accuracies but are generally not interpretable and do not explicitly quantify uncertainty, which should be the basis for real-time, risk-aware decision-making in intelligent transportation systems. Moreover, few efforts have been made in the robustness testing of models across heterogeneous traffic regimes, and few studies have practically addressed incomplete or noisy sensor data. Thus, there is still a need for an interpretable, theory-informed, and uncertainty-aware ensemble framework that draws from real ITS data to bolster predictive performance and forecasting transparency in expressway traffic.

## 3. Data and Methodology

### 3.1. Preprocessing and Time Alignment

Five temporally synchronized datasets were integrated into one feature matrix for supervised learning: *(i) Filtered_Data*, which had normalized congestion indices (traffic_index) assumed to be main ground truth labels; *(ii) Chronograf.xlsx*, with 18 feature measurements taken from the sensors (speed, density, flow, occupancy, and class-specific counts); and *(iii–v)* three sets of model-informed features (*Processed_CTM_Results*, *Processed_Helbing_Features*, and *Processed_Three_Phased_Results*) considered macroscopic traffic states, microscopic behavioural dynamics, and phase-transition probabilities, respectively. The *Filtered_Data* and *Chronograf.xlsx* were collected from the ITS sensors along the Chalong Rat Expressway by the EXAT. The *Chronograf.xlsx* dataset was accessed in November 2024, with the data recorded from January 2023 up until June 2024. The *Filtered_Data* dataset was accessed in January 2025, covering periods from February 2023 to April 2024. A summary of the measurements, variables, sources, and units for each of the datasets is provided in Table 1. All datasets were aligned with a common timestamp in UTC+7 and merged on the segment identifier (id_chunk) corresponding to actual expressway sections. For the *Chronograf.xslx* dataset, 15–20% of the missing values were imputed to preserve continuity, while sparsity in CTM and three-phase features was allowed since the traffic index was the primary supervisory signal.

To guarantee data quality and synchronize time series with the chronology of the Chronograf.xlsx dataset, several different measures were carried out. Most of the missing values were caused by short sensor dropouts or communication delays, which usually lasted a few seconds and, in exceptional cases, a few minutes. A total of 3299 missing values were noted, which amounted to approximately 15.78% of the entire dataset. To fill in these gaps, two imputation methods were employed. The forward-fill interpolation method was chosen for missing parts of ≤5 min to maintain continuity. Meanwhile, for longer or irregular gaps, a ±5 min rolling mean smoothing technique was used to lessen noise while still reflecting local trends. If any sensor-day combination had more than 10% data missing, that data slice was not included in training to prevent bias. Eventually, all time series were resampled to 5 min intervals to be compatible with external variables and to achieve a uniform timeline throughout the datasets.

### 3.2. Methodology Framework

**Figure 2:**
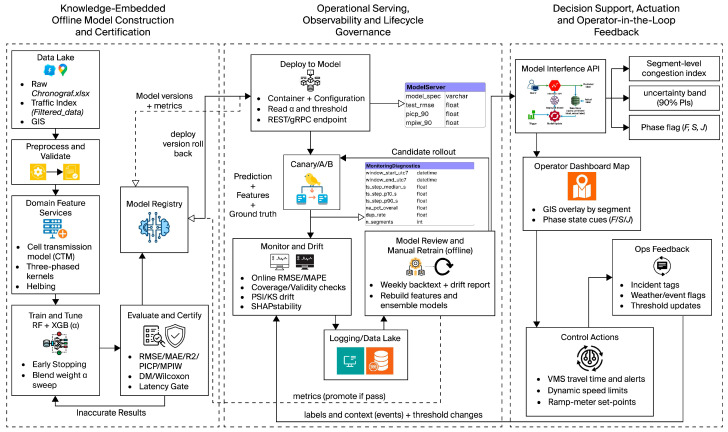
End-to-end framework for theory-guided ensemble learning in real-time congestion forecasting on the Chalong Rat Expressway. The solid arrow illustrates the main forward workflow and the dashed arrow points to monitoring and feedback processes.

This methodological framework (Figure 2) combines traffic theory principles with machine learning to produce a forecast-and-decision-support system that caters to expressway operations in a closed feedback loop. The system begins with a knowledge-embedded offline phase that preprocesses raw sensor and traffic data, enriches these datasets with theory-derived features, and subjects them to ensemble model training under strict certification criteria. To preserve reliability in the face of changing traffic dynamics, the operational layer controls deployment, run-time monitoring, and adaptive retraining of ensemble models based on drift diagnostics and validations controlled under strict versioning. Predictive outputs are finally tied into real-world controls through decision support systems that provide congestion indices, recommendations on phase states, and corresponding certainty bands to traffic controllers. This formalization is a linking of the theoretical model to a widely deployed infrastructure approach under which interpretability and robustness in real-time traffic management are reached.

All the mathematical formulations that were used in specifying the traffic-theoretic characteristics, including macroscopic flow dynamics (CTM), Kerner’s phase transition surrogates, Helbing’s microscopic behaviour models, ensemble design, and uncertainty quantification, are elaborated in detail at the end of the document in Appendix A.

### 3.3. Formalization of Predictive Modelling and Uncertainty Estimation

This section supplements the conceptual framework presented in the previous one by giving the formal mathematical structure that the proposed ensemble modelling pipeline is based on. The hybrid forecast model has incorporated temporal dependencies, ensemble blending strategies, and calibrated uncertainty quantification, thus providing robust and interpretable predictions of real-time traffic congestion.

#### 3.3.1. Temporal Backbone and Ensemble

Temporal dependencies were modelled using an AutoRegressive with eXogenous input (ARX) structure to ensure the use of only past information and rolling-window statistics to prevent information leakage. The general ARX representation is

(1) $$y_{t}=\sum_{l\in L}\emptyset_{l}y_{t-l}+\sum_{k}\beta_{k}x_{k,t-l}+\varepsilon_{t}\;$$

The lag set L consists of the fixed lags and the rolling-window statistics that ensure the stabilization of short-term dynamics within the autoregressive framework. Such a framework became the basis of the Hybrid-AR (safe) variant, where time modelling can be considered more robust in a real-time forecasting setting [28]. Furthermore, the temporal backbone was fused with tree-based ensembles by convex blending of random forest and XGBoost predictors for further accuracy enhancement. The ensemble prediction is

(2) $${\hat{y}}_{t}^{\left(ens\right)}=\omega {\hat{y}}_{t}^{\left(XGB\right)}+\left(1-\omega \right){\hat{y}}_{t}^{\left(RF\right)},\;\;\;\;\;0\leq \omega \leq 1\;$$

This blending weight ω was deliberately tuned for the metrics on validation error, such as the RMSE. This method utilizes the complementary strengths of the two predictors, where XGBoost is sensitive to nonlinear feature interactions, while random forest develops robustness against variance. These two methods were not singled out because they are implemented into Scikit Learn or are aligned with the Python ecosystem, but rather were chosen because boosting and bagging techniques are considered the most popular and widely presented ensemble learning techniques. It has been recognized that they hold a good balance between accuracy improvement and interpretability of the ensemble type [4]. Both models support deriving feature importance, scaling up to large datasets, and integration with SHAP analysis thereby making them the best fit for intuitive, uncertainty-aware forecasting frameworks for real-time operations. According to the theory in forecast combination, knowingly exploiting model diversity in this manner reduces generalization error [29].

#### 3.3.2. Distributional Prediction and Calibrated Uncertainty

Pinball (quantile) loss for level τ∈ (0, 1), is expressed as follows:

(3) $$L_{t}=\sum_{t}\rho_{\tau }(y_{t}-q_{\tau }\left(x_{t}\right))\;\;\;\;\;\;,\;\;\;\;\rho_{t}\left(u\right)=u[\tau -1_{\left\{u<0\right\}}]\;\;$$

In quantile regression forests, separate gradient-boosted trees are used for the lower and upper quantiles, conventionally at τ = 0.05 and τ = 0.95. These trees form approximations of conditional quantile functions, ${\hat{q}}_{0.05}(x)$ and ${\hat{q}}_{0.95}(x)$, which determine the lower and upper bounds of the prediction interval [30].

In addition, we conformalized quantile regression (CQR) corkscrews into the standard quantile regression by calibrating residuals on an independent validation set to guarantee coverage. For every instance, the rule states that a nonconformity score is assigned as

(4) $$s_{t}=max\{{\hat{q}}_{0.05}\left(x\right)-y_{t}\;\;\;,\;\;\;y_{t}-{\hat{q}}_{0.95}\left(x\right)\;,\;\;0\}$$

taking $\left(1-\alpha \right)$, which is the quantile ${\hat{s}}_{1-\alpha }$ of {$s_{t}$}, and forming a valid forecasting band as follows:

(5) $$\left[{\hat{q}}_{0.05}\left(x\right)-{\hat{s}}_{1-\alpha }\;\;\;,\;\;\;{\hat{q}}_{0.95}\left(x\right)+{\hat{s}}_{1-\alpha }\;\right]$$

Through adjustment, prediction intervals are ensured to attain the nominal coverage desired (80%, 90%, or 95%) under minimal distributional assumptions, directly supporting the reported uncertainty metrics in the results [31].

#### 3.3.3. Statistical Significance, Interpretability, and Drift Diagnosis

Model performance was treated through a dependence-aware moving-block bootstrap to cope with temporal autocorrelation. Confidence bounds for RMSE differences between models were constructed as follows:

(6) $$∆={RMSE}_{\left(Blend\right)}-{RMSE}_{\left(Persist\right)},\;\;\;\;\;\;{\;CI}_{95\%}=[{∆}^{0.025}\;,\;{∆}^{0.975}]\;$$

We aimed to verify whether the blended ensemble’s improvement over the persistence baseline was statistically robust and not just attributable to noise [32].

For interpretability purposes, TreeSHAP attributions were applied to the final ensemble [33]. Important predictors—including the congestion effect, vehicle class counts, rolling traffic statistics, and mean space headway—corresponded with traffic flow theory and supported the plausibility of learned relationships.

### 3.4. Model Training, Validation, and Performance Evaluation

**Figure 3:**
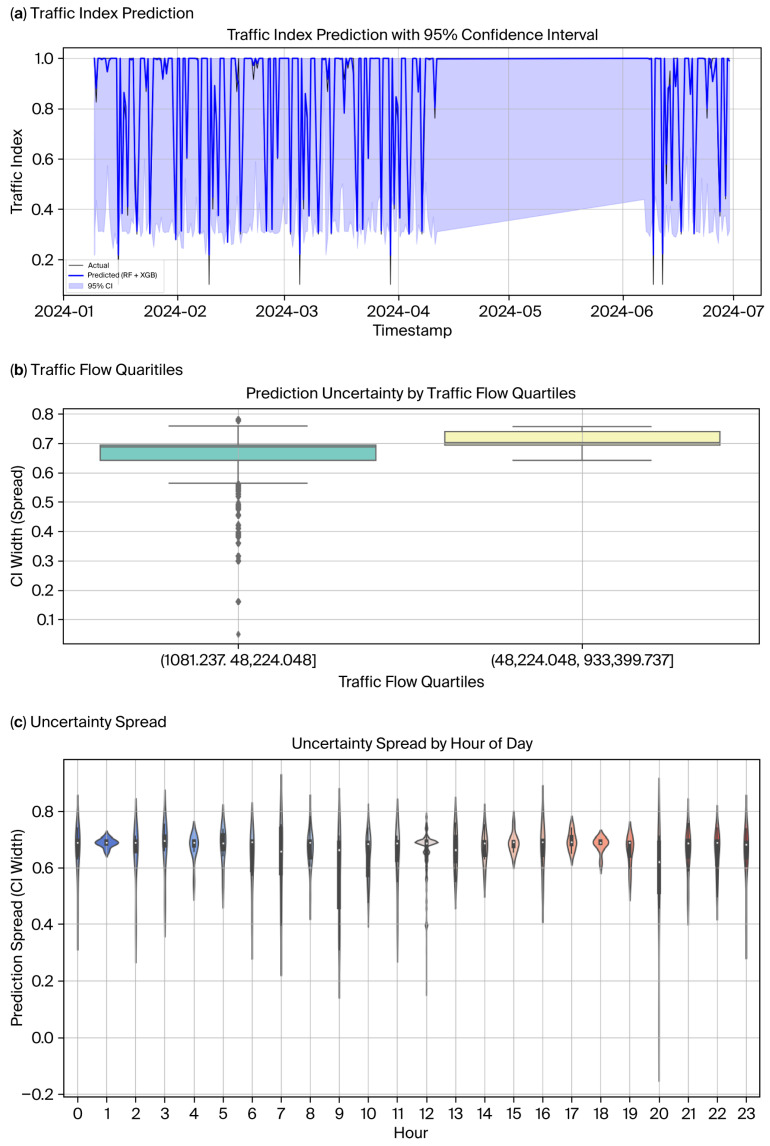
Error and uncertainty diagnosis across candidate models. (**a**) Time-series prediction of traffic index with 95% confidence intervals, displaying agreement between observed and hybrid RF–XGB forecasts. (**b**) Predictive uncertainty accounted for depending on traffic flow quartiles, emphasizing higher variances in congested zones. (**c**) Diurnal cycling and hourly structure of the spatiotemporal spread of uncertainty from peak awakening hours.

To maintain consistent evaluation standards, the models were trained using data divided into sequential time-based segments and then tested on a separate reserved test period, with the optimal model parameters determined based on validation results from a separate validation subset. All models were implemented in Python 3.10 through the scikit-learn (v1.3. 0) and XGBoost (v1.7. 6) libraries. The implementation of SHAP values was achieved with the help of the SHAP Python package (v0.42. 1), whereas conformal prediction intervals were estimated with MAPIE. Data processing was taken care of using pandas and NumPy, and all experiments were performed in a Jupyter notebook executed on a Linux-based workstation (Intel Core i9 CPU, 64 GB RAM). No PyTorch or GPU accelerated frames were used in this study, considering all of them to be interpretable ensemble models for structured traffic data. Validation methods such as blocked and rolling-origin techniques were employed to address temporal leakage and maintain dependence in the data [34,35]. The dataset composition and sampling intervals are detailed in Table 2, with error diagnostics displayed in Figure 3a–c for multiple candidate models, revealing systematic residual structures that emphasize the need for ensemble stabilization. From April to July, the elevated unpredictability was associated with wider confidence intervals, as shown in Figure 3a, which is in line with the Songkran festival as a surge event, with abnormally high traffic flows [36].

**Figure 4:**
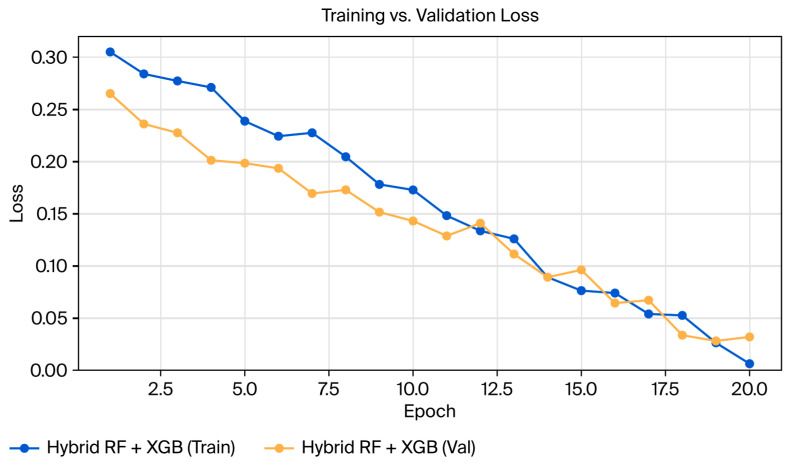
Training and validation loss curves for the hybrid random forest–XGBoost ensemble.

The ensemble configuration involved random forest and XGBoost regressors combined through a weighted convex blend, with the blending parameter selected to minimize validation RMSE [37]. An additional persistence blending coefficient was incorporated to stabilize predictions near flow transitions to produce the final hybrid model. Details of the full hyperparameter settings are provided in Table 3, while Figure 4 interprets the convergence of training and validation loss to select the optimal blending configuration while achieving minimal validation error under blocked cross-validation [4].

To evaluate the performance of the model, both point prediction and uncertainty quantification metrics were used as a set. Specifically, the mean absolute error (MAE), root mean square error (RMSE), and coefficient of determination (R^2^) mainly assessed prediction accuracy. MAE measured the average error magnitude, RMSE measured larger errors, and R^2^ quantified the percentage of variance explained by this model. The predictive interval coverage probability (PICP) and mean predictive interval width (MPIW) from the conformal prediction are additional ways to check the quality of uncertainty estimates. PICP marks the percentage of true y-values lying in the predicted interval, and MPIW measures the sharpness of the interval. These combined metrics were selected to explain not only how precisely the forecast had been executed but also how confidently and consistently the model quantified uncertainty under changing traffic conditions.

## 4. Results

The results from the evaluation are presented to examine the accuracy, robustness, and uncertainty features of the proposed ensemble approach when applied to the Chalong Rat Expressway dataset. Performance was analysed at multiple levels, from general error metrics and residual diagnostics to robustness analyses under different traffic regimes and levels of congestion. Uncertainty quantification was performed via a set of calibrated prediction intervals, and statistical testing was undertaken to establish the significance of identified improvements. Together, the results represent a complete evaluation of both accuracy and reliability in real-time congestion forecasting.

### 4.1. Overall Performance and Classification

Details regarding the validation and testing data obtained for the different models are provided in Table 4, which can be defined as an ablation study, where architecture components in the proposed hybrid ensemble were involved are not involved individually to test their contributions, especially the table incorporating the hybrid ensemble (Final Blend), its constrained form (Hybrid-AR safe), the archetypal persistence model, and the exogenous-only hybrid model. The Final Blend model achieved the lowest test RMSE value of 0.045752 and the highest R^2^ value of 0.889421 compared to the two baselines, showing that it has greater predictive strength. Though the persistence model achieved a slightly lower MAE, the higher RMSE eroded explicative strength, highlighting the intricacies involved in accurately identifying changes. On average, it greatly outperformed the exogenous hybrid model in almost all evaluation metrics, emphasizing the significance of introducing autoregressive dynamics.

**Figure 5:**
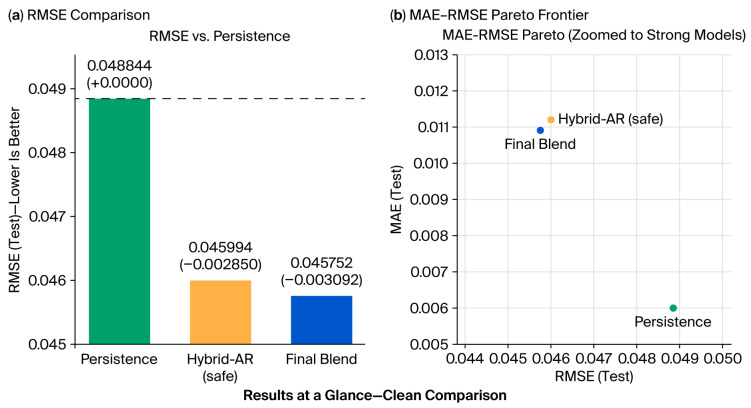
RMSE and MAE–RMSE comparison demonstrating Final Blend outperforming Hybrid-AR and persistence: (**a**) RMSE comparison of candidate models against persistence baseline; (**b**) MAE–RMSE Pareto frontier (zoomed in to high-performing models).

**Figure 6:**
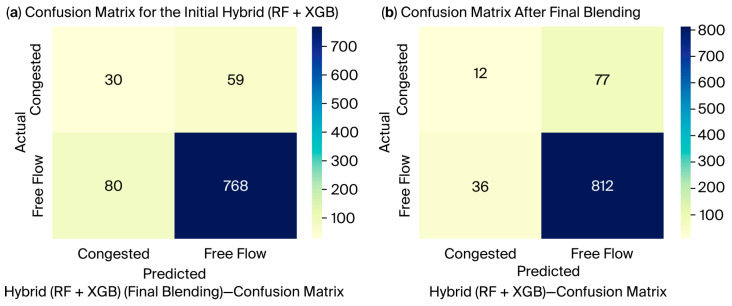
Comparison of hybrid (RF + XGB) model performance before and after final blending using confusion matrices: (**a**) confusion matrix for the initial hybrid (RF + XGB) model showing poor recall for the congested class; (**b**) confusion matrix after final blending, showing improved detection of congested cases.

These outputs are reinforced by Figure 5a, which illustrates a comparison of RMSEs with respect to persistence, and Figure 5b, which reveals the Pareto frontier of the MAE vs. RMSE. Both figures primarily show that when using the Final Blend model, a reduction in error could be achieved without significant effects on stability. Consequently, an outstanding characteristic is illustrated in Figure 6a,b through confusion matrices that pertain to the classification performance between congestion and free flow. The blend of these ensembles decreased the misclassification of the congestion states by the initial ensemble model, highlighting the effectiveness of blending in capturing those nonlinear transitions between traffic regimes.

The outcomes confirm that the Final Blend model provided the most effective balance between accuracy and stability. It has lower errors than the other models, maintained strong explanatory power across regimes, and minimized the misclassification of congested states. This shows that blending improves not only numerical accuracy but also the reliability of predictions with changing traffic conditions.

The reliability of traffic forecasting models’ inputs is well enhanced by the quality of the collected data. Sensor readings from the *Chronograf.xlsx* and *Filtered_data* datasets that were used in this research may be in some cases corrupted due to some hardware problems and transmission errors, which have the potential to affect the proposed forecast of delayed or inaccurate traffic. Even though imputation was used to address missing values, noisy or biased sensed data can still feed through into features and affect model performance. Some of the inputs from the model can be robust in their ensemble methods against the evidential impacts of many input streams [6]. However, data degradation may vary, for example coming from the case of fragile sensors, may occur in important locations. For instance, merge points or bottlenecks might have serious repercussion on the predictive capabilities of models. Future research could experiment with improving data gleaning approaches by proposing a sensor confidence score, taking it as a relative term during training of the model, or even using various schemes that are aware of reliability during modelling to become very certain about specific handling aspects related to data noise and the constraints of the sensor [8]. Additionally, some explicitly dedicated preprocessing and imputation may play a big part in improving the reliability of information on detectors, particularly in circumstances involving dropouts or erroneous values from loop detectors [26].

### 4.2. Robustness and Error Analysis

Traffic behaviour presents significant heterogeneities in different temporal and congestion states, requiring a dedicated methodology to successfully validate the generalization capability across entire forecasting horizons [10]. To capture these effects, the models were segregated by peak and off-peak times which characterized those impacts, with high-congestion conditions being compared to low congestion. Therefore, it was necessary to measure predictive stability against demand shifts and structural switches in flow behaviour.

Performance stratification during peak and off-peak periods (Table 5) shows that the Final Blend and hybrid (constrained) models remained true to their accuracy, with Final Blend being slightly better during off-peak traffic with a slightly lower RMSE, and the hybrid model performing slightly better in peak traffic. This means that the blending ensemble was more stable under smoother traffic dynamics, whereas the constrained hybrid model stayed resilient to transient demand spikes.

A complementary pattern emerged when the results were grouped by congestion severity (Table 6). Under high congestion, the hybrid model achieved slightly lower error than Final Blend, suggesting better adaptation to unstable conditions. In contrast, Final Blend showed better performance under low-congestion conditions, where traffic patterns were less volatile. Together, these findings suggest that the two models captured different aspects of traffic variability and that blending provided a balance of accuracy for different operational regimes that pose distinct challenges. In addition to the robustness comparisons, the statistical significance testing and residual diagnostics described in Appendix B lend support to the findings, confirming that the differences in performance observed were not only statistically valid but also stable over time.

**Figure 7:**
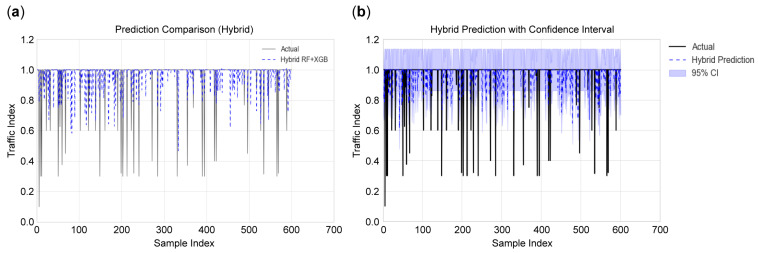
Visual comparison of the ground truth and predicted traffic index under varying conditions: (**a**) Comparison between the actual traffic index and predicted values from the hybrid RF + XGB model, illustrating the model’s ability to track congestion patterns across various conditions; (**b**) hybrid model predictions with 95% confidence intervals, demonstrating uncertainty quantification and identifying periods of prediction deviation.

The model’s behaviour in different traffic scenarios was visualized by plotting predicted and actual congestion levels for the hybrid model. In Figure 7a, the observed value is overlaid on the predicted traffic index, showing that the model captured the congestion trend well during regular scenarios. Correspondingly, Figure 7b shows the same predictions but with uncertainty bounds at the 95% confidence level, reacting to the increase or decrease in traffic flow. Collectively, the plots help identify periods of heightened prediction higher than normal performance, as well as periods of underperformance, especially when fast congestion drops reveal prediction lags. These types of visual analysis complement quantitative measures by reflecting on the localized behaviour of the model over time, thereby serving a pragmatic purpose in deployments by suggesting situations in which the model output should be interpreted with caution.

**Figure 8:**
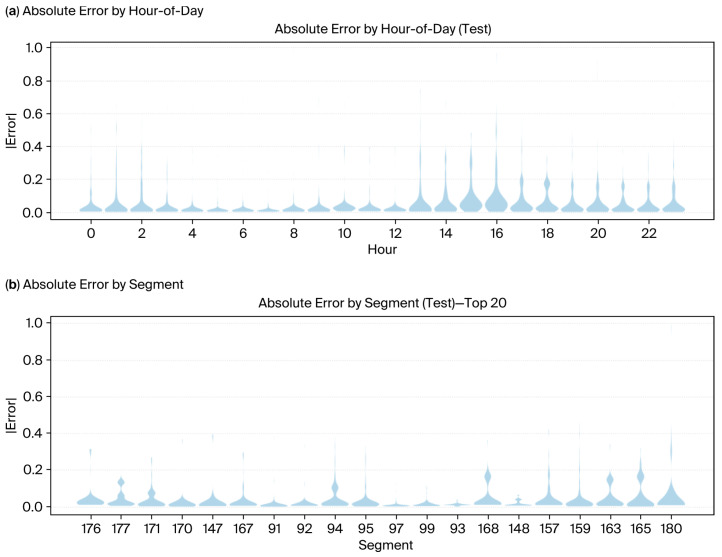
Error distribution analysis across temporal and spatial dimensions on the test set before blending. (**a**) Absolute error distribution by hour of day; (**b**) error distribution by the relevant segment.

**Figure 9:**
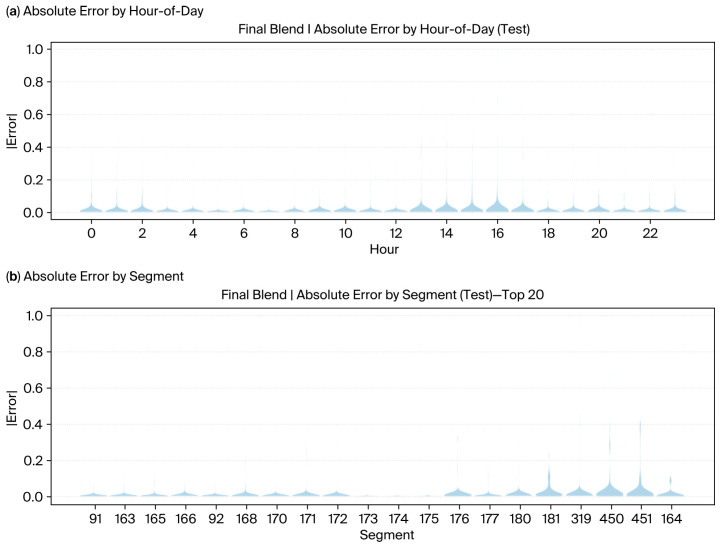
Error distribution analysis across temporal and spatial dimensions on the test set after blending. (**a**) Absolute error distribution by hour of day; (**b**) error distribution by the relevant segment.

The violin plots shown below (Figure 8a,b) complement the formation of errors by hour of day and road segment, revealing that errors were concentrated during late afternoon peaks and caused a bottleneck in specific segments. When contrasted against Final Blend (Figure 9a,b), the visibly diminished error spreads once again reinforce its role in uncertainty reduction and prediction stabilization amidst heterogeneous traffic settings.

Consequently, regime-aware stratification was shown to be crucial for evaluating of performance, with Final Blend being the overall more balanced approach, although it is contingent upon the complementary strengths of constrained hybrid models for greater adaptive, context-specific deployment options.

### 4.3. Uncertainty Quantification and Calibration

Uncertainty quantification was performed to safeguard the reliability of predictive intervals and to ensure good calibration under various traffic regimes. Conformal prediction intervals were computed over the test set to obtain the PICP and MPIW scores.

There was a near similarity in the PICP and MPIW values across different α-levels and traffic regimes from conformal predictions, as shown in Table 7 and Table 8. This is because a single global calibration set was used, resulting in uniform non-conformity scores that enforced marginal coverage for all segments. While this guaranteed validity at the population level, it may have masked the conditional variation between different traffic states. The observed resemblance matches the theoretical findings that conformal prediction ensures unconditional validity but globally calibrates it away in the presence of heteroscedastic effects and regime-specific variability [38].

In particular, the results show how the conformal prediction intervals were uniformly reliable in both the peak and off-peak periods. Empirical coverage equalled approximately 93%, with minor variation between regimes when considered at the nominal 95% level. The width interval remained stable across α thresholds, suggesting that the method produced uncertainty bands independent of weather conditions. Coverage was slightly higher than nominal during peak hours, tilting the coverage to conservativeness during congestion. However, coverage in off-peak intervals tended to hit nominal targets more closely.

**Figure 10:**
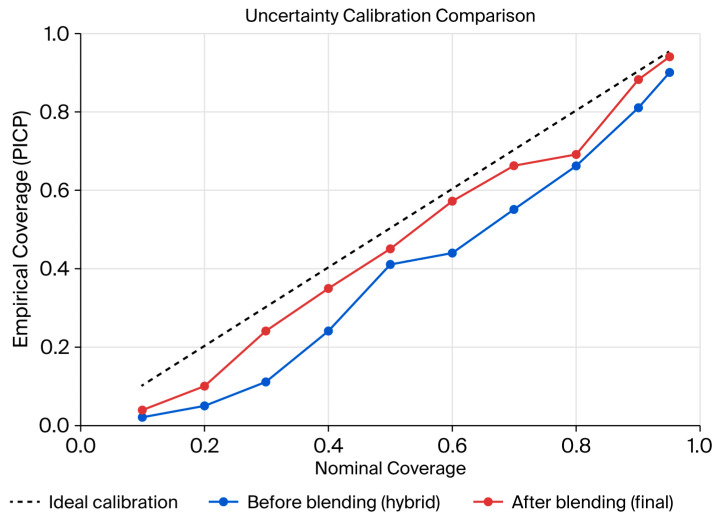
Uncertainty calibration comparison between the hybrid (before blending) and Final Blend models, showing empirical coverage (PICP) against nominal coverage.

The calibration plots in Figure 10 provide further evidence of these findings. For both the hybrid and Final Blend models, empirical coverage increased monotonically with nominal coverage perfectly along the diagonal when considering an ideal calibration scenario. Since both models were valid, Final Blend tended to stay closer to the diagonal through most confidence levels, especially in the area with mid-to-high coverage. This might imply that blending accomplished not only increased point forecasting stability but also increased reliability of predictive intervals, which in turn produced uncertainty estimates that were better calibrated to the actual underlying data distribution.

The uncertainty analysis confirmed that prediction intervals maintained steady coverage under various traffic regimes and consistent reliability at times of varying demand. Calibrating the method at the global level compromised the ability to reflect the fine variability that occurs in peak and off-peak states. More importantly, blending improved calibration, since blended intervals very closely matched the theoretical probability of coverage and therefore must be more robust in the face of wide-spectrum traffic dynamics.

### 4.4. Interpretability and Feature Contributions

Feature importance analysis was performed to evaluate the different modelling strategies distributing explanatory power across the input space. The random forest showed a very concentrated importance pattern, while XGBoost distributed importance evenly among variables. The SHAP-based hybrid ensemble, in contrast, communicated richer attributions with respect to macroscopic traffic states and microscopic behavioural dynamics. Aggregating SHAP values by the traffic theory domain revealed how the three distinct theoretical frameworks contributed to model predictions differently.

**Figure 11:**
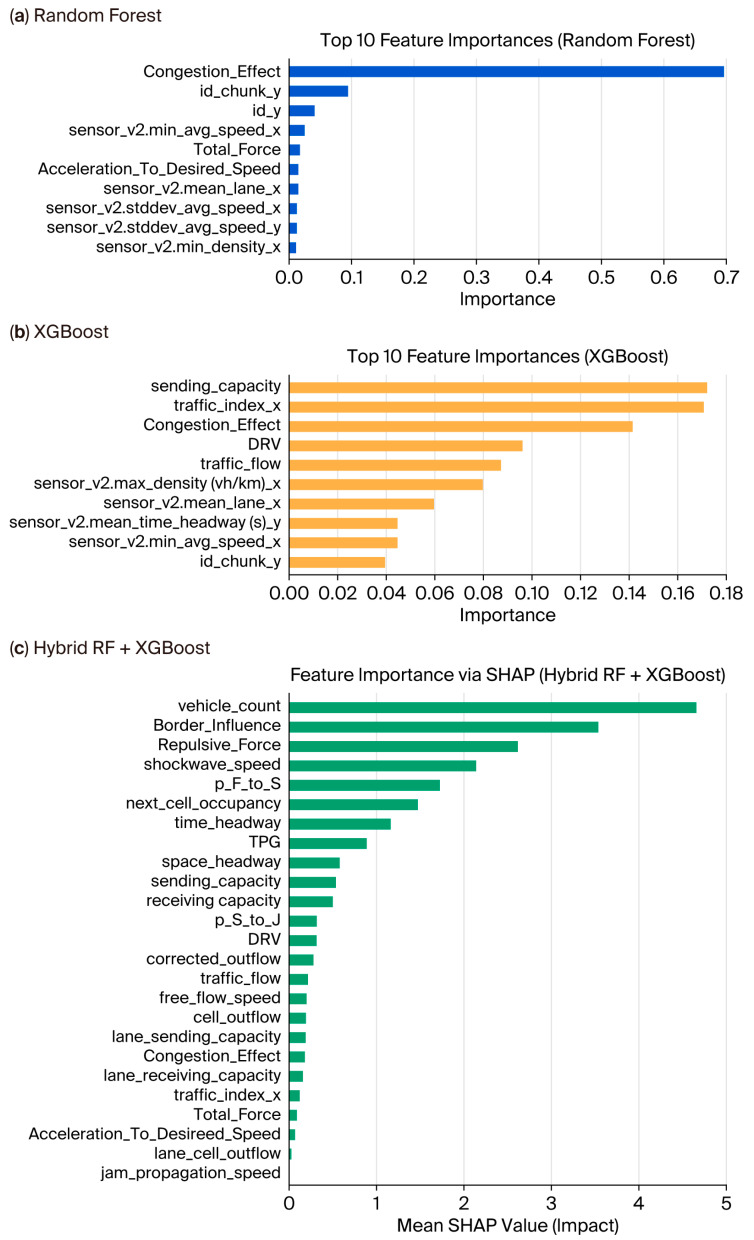
Feature importance analysis. (**a**) Random forest with a dominant reliance on the congestion effect; (**b**) XGBoost with balanced importance across traffic capacity and flow-related indices; (**c**) SHAP-based hybrid model capturing both microscopic and macroscopic traffic dynamics.

The random forest (Figure 11a) observed a very sharply concentrated dependency on the congestion effect variable, with other descriptors very rarely contributing, including lane-level averages and density measures. This suggests the tendency of tree bagging methods to outweigh single, dominant factors or explanatory variables. Conversely, XGBoost (Figure 11b) distributed importance among variables, resulting in congestion descriptors, flow, and density metrics being almost equal. The hybrid ensemble analysed by SHAP (Figure 11c) offered an even more interpretable picture of feature influence regarding magnitude and direction of impact. Vehicle count, border influence, repulsive force, and headway emerged as the most influential drivers, followed by combinations of microscopic indicators (headway and shockwave speed) and macroscopic traffic states. This deep attribution brought out SHAP’s ability to reveal multi-scale interactions beyond those captured by individual models.

**Figure 12:**
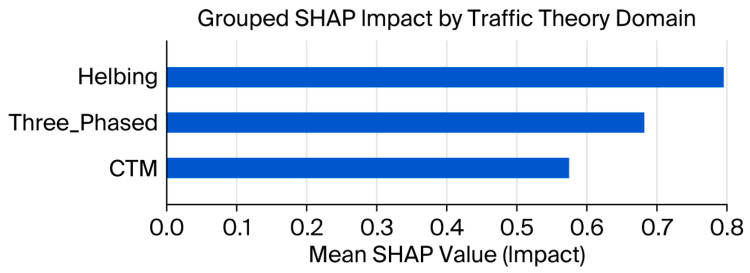
Grouped SHAP impacts aggregated by traffic theory domain, showing the relative contributions of the Helbing, three-phased, and CTM descriptors.

When SHAP values were aggregated by traffic theory domain, as shown in Figure 12, a clear hierarchy of contributions emerged. The Helbing model’s behavioural descriptors clearly dominated, revealing the importance of microscopic interactions in shaping predictive outcomes. The traffic features of the three-phase variety had a significant but secondary influence, while the CTM-based measures were less important in comparison. This stratification points to the hybrid model’s ability to capture multi-scale dynamics, thereby enhancing instead of diminishing interpretability by associating learned importance with established theoretical paradigms in traffic flow modelling.

The findings jointly indicate that random forest possibly tends to emphasize a single variable, whereas XGBoost balances its effects among capacity indices and congestion indices, and the hybrid SHAP framework fuses multi-scale traffic descriptors. This explanation framework diversity is what supports the strong generalization and robustness of the hybrid ensemble.

## 5. Discussion

### 5.1. Interpretation of Findings

Stratified evaluation further confirmed that off-peak hours when combined with the ensemble, resulted in a lower RMSE than persistence, whereas the hybrid model maintained robustness during the peak demand period, acting in a complementary manner. The prediction intervals generated by conformalized quantile regression adaptively widened during unstable regimes, demonstrating the method’s ability to account for traffic heteroscedasticity [39]. Importantly, the similar coverage values, regardless of the regime or α levels, were a testament both to the strengths and limitations of global calibration: It has strong marginal validity but minimal conditional sensitivity. This evidence weighs strongly in favour of running traffic theory as a stabilizing influence, since the theory itself imparts an additional structure that compensates for the uniformity of calibration. Though these deep-ensemble approaches [6] tend to have better predictive performance and yet diminished interpretability, the hybrid model is intended to strike a balance as it comes with calibrated uncertainty intervals while remaining interpretable via SHAP analysis. While earlier studies regarding the Chalong Rat Expressway used simulated incident data [3], the model described here uses real-world ITS sensor feeds obtained from EXAT for practical deployment. In contrast to tree-based traffic forecasting, which integrates theoretical aspects in neither design nor approach [4], the nature of existence-informed feature design is more grounded with respect to micro- and macro-scale dynamics (such as CTM, Kerner’s theory, and Helbing force’s model), lending it the property of being forecastable in the vicinity of congestion transitions and thereby making it more robust. Thus, it contributes to the progress of research by incorporating interpretability, realistic calibration, and operational readiness, which were often overlooked in prior models.

Interpretability analysis presented yet more evidence for the working hypothesis. While the random forest paradigm tended to concentrate the predictive importance into a single, dominating feature, XGBoost distributed importance more diffusely across macroscopic and microscopic indicators. Meanwhile, SHAP-based analyses of the hybrid model showed a far richer explanatory structure. Grouping the SHAP values via traffic theory domains showed that the Helbing-type behavioural surrogates took the lead, followed by the three-phased descriptors; CTM variables had their contributions in the middle. This alignment with theory helped validate the feature engineering approach and showed that interpretable ensembles can simultaneously stay rooted in established traffic flow paradigms and score high on predictive fidelity.

The integration of traffic theory with machine learning in the proposed structure directly addresses the three identified key limitations in the literature. The focus of earlier studies has mostly been on black-box models like LSTM, GNN, and ensemble techniques [6,10,11,17], while the incorporation of traffic domain knowledge has been very limited [6,8,10,11]. Just a handful of research works in the recent past have tried to tackle interpretability with the help of model explanation frameworks like SHAP [33], whereas the majority do not provide physically meaningful outputs that are helpful for decision making [11,16]. First, this approach avoids the utilization of black-box models that in most instances hamper interpretability or transparency, though they offer high predictive accuracy. This leaves traffic forecasting efforts tied up in black boxes, which keep the actual phenomena of the underlying traffic dynamics or derived control actionable strategies [4,5]. By including variables such as the repulsive force, jam propagation, and critical density captured in traffic theory into the feature set and model constraints, the structure conveys a physically meaningful structure that enhances interpretability and supports operational decision-making. Second, the absence of physical constraints or domain knowledge in many existing models normally leads to poor generalization across different traffic conditions [6,8]. This methodology addresses this issue through embedding a bias of traffic theory as a form of induction, thereby transforming the system into a robust and high-performing one under different traffic conditions. Third, the interpretation of physically based output variables provides a direct insight for traffic operators, bridging the gap between prediction and intervention in practice–a limitation that is largely unaddressed in much of the existing literature.

The results of this research are in line with the modelling difficulties experienced in other developing urban areas, where the traffic behaviour, infrastructural constraints, and lack of data are quite different from the situation in highly equipped environments. In the case of the Nairobi Metropolitan Area, machine learning models have been built that utilize very few sensor data along with exogenous variables to predict congestion even with limited detector coverage [40]. Furthermore, in the case of New Delhi, India, forecasting methods that depend on weather and time features have proved to be successful even under restricted data conditions using support vector regression [41]. By showing the use of data-driven models in complex and low-resource urban systems, these examples not only highlight the need for local calibration but also show the full flexibility of these models. The importance of such comparative insights bolsters the transferability potential of the proposed framework and situates this research within the wider literature on congestion forecasting in emerging urban contexts.

### 5.2. Implications and Future Works

The operational aspect implies that the results are directly relevant to traffic management on an almost real-time basis for the Chalong Rat Expressway and other similar urban tollway systems. Accurate point forecasts, coupled with calibrated uncertainty bounds, offer decision-makers certainty and a degree of doubt. These interventions range from adaptive ramp metering to speed harmonization and congestion warnings. The very incorporation of features informed by theory guarantees that the outputs of the system are interpretable, an outright axiom for building trust in Intelligent Transportation Systems. Tracing the explanatory power of forecasts back to the concept of shockwave propagation or driver response dynamics aids in fostering transparency and thereby strikes a balance between black-box algorithms and traffic engineering practice.

Beyond technical performance, such a model has implications for actionable urban mobility planning. Real-time, high-resolution traffic forecasting and tracking that is demonstrated by the Aimsun system for Bangkok [9] can become the basis for adaptive traffic management strategies such as those that involve varying lane assignments, changing speed limits, and implementing new congestion pricing. This practice reduces travel time per unit and fits in with broader policy goals like reducing fuel consumption and emissions in line with sustainability targets [6]. Additionally, using interpretable AI methods [4,5] saves the black box, leading to the development of trust among the stakeholders and raising acceptance from the public, thus fulfilling the essential prerequisites for publicly participating in and reviewing planning issues. Overall, the concept supports policy-relevant approaches to mitigating congestion and developing an urban mobility strategy that goes on for a long period.

However, limitations may arise. The single global calibration dataset provided marginal validity but masked conditional variability across traffic regimes, which, in extreme cases, may lead to a reduction in its reliability. Another drawback could be the corridor-specific evaluation, which showed robust results for Chalong Rat but cannot yet guarantee transferability to other expressways of different geometries, driver populations, or sensor ecosystems. Nonetheless, internal transferability was found only moderately within the lane. As illustrated in Table 5 and Table 6, the hybrid and Final Blend models achieved relatively consistent performance during peak/off-peak times and congestion levels. Such robustness across traffic regimes implies the generic capture of traffic patterns by the proposed framework, even within a heterogeneous local environment. While such data are insufficient without multiple corridor validations, this at least supports the capability of the model architecture to adapt to structurally different environments. Future work should validate the model’s generalizability through cross-location testing and simulated transfer scenarios. These limitations point to the need for further methodological refinements and broader validation.

Future studies should investigate conditional and group-wise conformal calibration, which could retain the advantages of marginal validity but still consider the different variances among traffic regimes. However, the possibility of adaptive ensemble weighting is another research avenue where blending coefficients could be driven by real-time indicators of flow instability, thus allowing the model to adapt better to the prevailing conditions. The validation process should also cover testing the theory-informed features’ generalizability in more diverse settings using Bangkok’s multi-corridor networks as well as other comparable megacities.

Taken together, adaptive calibration, dynamic blending, and wider cross-network validation have the potential to elevate the framework from corridor-specific optimization to the ability of large-scale and deployable forecasting solutions. However, the present model does not consider urban form or land use characteristics, which are increasingly acknowledged as crucial factors in traffic demand. There is considerable evidence that built environment factors, such as density, land use diversity, and street network design, have a considerable impact on trip generation and congestion levels [42,43]. Meta-analytical studies assert that compact, mixed-use environments with good connectivity are linked to lower vehicle travel and greater use of different modes of transport [43]. Additionally, larger-scale urban analyses show that the layout and amount of land used have a significant impact on determining the patterns of traffic congestion [44]. The integration of these spatial indicators into future models might result in better generalizability of the data and offer a more thorough understanding of the congestion heterogeneity in complicated urban areas. Real-world traffic management applications stand to benefit enormously from the interpretability of this model. In contrast to vacuum-like black boxes that eschew explanation, interpretable models allow traffic operators to track the forecast outputs down to such input features as the interactions of speed and density, congestion thresholds, or critical density from actual field measurements. Hence, transparency translates into informed and conscious decision-making, especially significant during incidents of adaptive signal controls or congestion pricing. Operators may require an understanding that downstream occupancy or the shockwave propagation variable induces bottlenecks so that they can initiate anticipatory control measures. This is evidenced by the data presented in Figure 8 and Figure 9, where the model was able to predict peak congestion in the morning and evening periods and detected differences in congestion levels between the main areas. There was a case when a considerable increase in the forecasted congestion was seen on the downstream area just before the evening peak, although the speeds were relatively stable—this was the situation of increased repulsive force and upstream congestion buildup. This model predicts risk based on traffic-theory variables and thus builds up trust, helps in making operational decisions, and makes it easier to adopt into an institution’s workflow where accountability of the model is a prerequisite [4].

## 6. Conclusions

This research developed a domain-theoretic ensemble framework to predict real-time traffic congestion along the Chalong Rat Expressway. By embedding surrogates of macroscopic, mesoscopic, and microscopic traffic theory within machine learning models, we attained better robustness in accuracy across the traffic regime, while the SHAP-based analysis allowed for interpretability. The hybrid random forest and XGBoost model showed consistent improvements over single models and persistence baselines, especially for congestion transitions. Conformal prediction-based uncertainty quantification further contributed to reliability by providing calibrated intervals that responded adaptively to heteroscedastic flow conditions. The Final Blend which performed the best, provided a mean absolute error (MAE) of 0.01086, a root mean squared error (RMSE) of 0.045752 and an R^2^ of 0.889421 based on the test data. On quantification of uncertainty posterior conformal prediction intervals (PCIPs), the results indicated that the Final Blend model achieved a PICP of 92.97% under a 95% nominal confidence level. Moreover, this result increased to 93.46% during peak-hour activities. This conclusion validates previous findings that the ensemble not only significantly boosts predictive accuracy but also delivers acceptable levels of calibration and interpretability of its uncertainty estimates with sound real-time forecasting abilities under practical conditions. Furthermore, the results highlight the benefits of combining theory-based features with contemporary ensemble methods for balancing predictive accuracy and operational significance. Overall, this framework provides an implementable foundation for intelligent traffic management systems with future opportunities for dynamic calibration and cross-network generalization on large-scale urban tollway environments.

## Figures and Tables

**Table 1:** Data sources, variable names, units, and brief descriptions used in the traffic modelling framework.

Source	Variable	Unit	Description
*Filtered_data*	id	-	Unique ID for each data record
	id_chunk	-	ID of road segment on the expressway
	traffic_index	-	Congestion level indicator
*Chronograf.xlsx*	time (+7 UTC)	date time	Timestamp in Indochina time
	sensor_v2.max_avg_speed	km/h	Maximum average vehicle speed
	sensor_v2.max_density	vh/km	Maximum vehicle density per lane
	sensor_v2.mean_avg_speed	km/h	Average vehicle speed
	sensor_v2.mean_density	vh/km	Average vehicle density per lane
	sensor_v2.mean_flow (vh/h)	vh/h	Vehicle flow per hour
	sensor_v2.mean_lane	-	Average lane index used
	sensor_v2.mean_occupancy	%	Average percentage of lane occupancy
	sensor_v2.mean_space_headway	Metre	Mean spacing between vehicles
	sensor_v2.mean_time_headway	Second	Mean time gap between vehicles
	sensor_v2.min_avg_speed	km/h	Minimum average speed
	sensor_v2.min_density	vh/km	Minimum vehicle density
	sensor_v2.stddev_avg_speed	km/h	Speed variability (standard deviation)
	sensor_v2.stddev_density	vh/km	Density variability (standard deviation)
	sensor_v2.sum_count_class1	count	Count of motorbikes (2-wheel vehicles)
	sensor_v2.sum_count_class2	count	Count of passenger cars
	sensor_v2.sum_count_class3	count	Count of small trucks (e.g., 6 wheels)
	sensor_v2.sum_count_class4	count	Count of large trucks (10 wheels and above)
Derived (CTM)	vehicle_count	count	Total number of vehicles detected
	sending_capacity	vh/h	Maximum vehicle outflow from a cell
	receiving_capacity	vh/h	Maximum vehicle inflow to a cell
	cell_outflow	vh/h	Actual vehicle outflow from a cell
	lane_sending_capacity	vh/h	Maximum outflow per lane
	lane_receiving_capacity	vh/h	Max inflow per lane
	lane_cell_outflow	vh/h	Vehicles leaving a specific cell lane segment
	free_flow_speed	km/h	Speed in ideal (uncongested) conditions
	corrected_outflow	vh/h	Adjusted outflow after capacity constraints
Derived (Kernel)	shockwave_speed	km/h	Speed of traffic shockwave propagation
	jam_propagation_speed	km/h	Speed of jam moving upstream
	p_F_to_S	Probability	Probability of free flow → synchronized transition
	p_S_to_J	Probability	Probability of synchronized flow → jam transition
Derived (Helbing)	Acceleration_To_Desired_Speed	km/h	Driver’s acceleration toward desired speed
	Repulsive_Force	N (Proxy)	Force maintaining safe distance from leading vehicle
	TPG	-	Traffic phase group indicator
	DRV	-	Driver behaviour variable used in modelling
	Border_Influence	-	Influence of road edges on vehicle movement
	Total_Force	N (Proxy)	Combined force from all dynamic effects
	Congestion_Effect	-	Impact of traffic congestion on driver dynamics

**Table 2:** Dataset sampling rates, splits, and counts (Bangkok local time).

Dataset	Period Covered	Median (s)	Train	Validation	Test	Sampling Frequency (Hz)
*Filtered_data.xlsx*	February 2023–April 2024	300.0	113,876	24,353	24,393	0.0033
*Chronograf.xlsx*	January 2023–June 2024	65,400	505	108	108	0.000015

**Table 3:** Hybrid model blending parameters for the ensemble.

Model	Key Parameters	Value
Random forest	n_estimators	800
	max_depth	16
	Min_samples_leaf	5
	max_features	Sqrt
	bootstrap	True
XG Boost	objective	reg:squarederror
	tree_method	hist
	max_depth	6
	eta	0.05
	subsample	0.85
	colsample_bytree	0.85
	reg_alpha	0.5
	reg_lampda	4.0
	best_iteration	156
	early_stopping_rounds	100

**Table 4:** Validation and test performance of candidate models. Metrics include mean absolute error (MAE), root mean square error (RMSE), and coefficient of determination (R^2^).

Set	Model	MAE	RMSE	R^2^
Validation	Final Blend (Pers + (1-) Hybrid-AR)	0.007263	0.034152	0.910922
	Hybrid—AR (safe)	0.007602	0.034188	0.910739
	Persistence	0.003873	0.037508	0.892557
	Hybrid (exogenous-only)	0.060445	0.156530	0.871206
Test	Final Blend (Pers + (1-) Hybrid-AR)	0.010860	0.045752	0.889421
	Hybrid—AR (safe)	0.011351	0.045994	0.888252
	Persistence	0.005969	0.048844	0.873973
	Hybrid (exogenous-only)	0.085516	0.184808	0.804216

**Table 5:** Performance stratified by peak vs. off-peak times (test set).

Model	Segment	n	MAE	RMSE	R^2^	RMSE vs. Persistence
Final Blend	Peak	660	0.059194	0.103935	0.879136	0.155673
Final Blend	Off-peak	1830	0.069895	0.124574	0.757467	0.077583
Hybrid (constrained)	Peak	660	0.054744	0.102139	0.884857	0.157469
Hybrid (constrained)	Off-peak	1830	0.069752	0.126372	0.757344	0.075785

**Table 6:** Robustness by congestion severity (test set).

Segment	Model	n	MAE	RMSE	R^2^	RMSE vs. Persistence
High	Final Blend	1697	0.044695	0.084842	0.293755	0.050306
High	Hybrid (constrained)	1697	0.047384	0.092128	0.300862	0.043020
Low	Final Blend	793	0.114916	0.171463	0.750287	0.162178
Low	Hybrid (constrained)	793	0.105126	0.165449	0.756323	0.168191

**Table 7:** Conformal prediction summary of the test set.

Alpha	Segment	N	PICP	MPIW
0.05	All	24,393	0.926085	0.155303
0.05	Peak	6468	0.934601	0.155303
0.05	Off-peak	17,925	0.923013	0.155303
0.10	All	24,393	0.926085	0.155303
0.10	Peak	6468	0.934601	0.155303
0.10	Off-peak	17,925	0.923013	0.155303
0.20	All	24,393	0.926085	0.155303
0.20	Peak	6468	0.934601	0.155303
0.20	Off-peak	17,925	0.923013	0.155303

**Table 8:** Conformal coverage for Final Blend with confidence levels of 80%, 90%, and 95%.

Alpha	Nominal	Segment	n	PICP	MPIW
0.05	0.95	All	2490	0.929719	0.435646
0.05	0.95	Off-peak	1830	0.920219	0.435646
0.05	0.95	Peak	660	0.956061	0.435646
0.10	0.90	All	2490	0.875904	0.276992
0.10	0.90	Off-peak	1830	0.857923	0.276992
0.10	0.90	Peak	660	0.925758	0.276992
0.20	0.80	All	2490	0.785141	0.165285
0.20	0.80	Off-peak	1830	0.781967	0.165285
0.20	0.80	Peak	660	0.793939	0.165285

## Data Availability

The data presented in this study are not publicly available due to privacy restrictions imposed by the data provider (EXAT). Further inquiries can be directed to the corresponding author.

## Abbreviations

The following abbreviations are used in this manuscript:

ARX	AutoRegressive with eXogenous input
CQR	Conformalized quantile regression
IDM	Intelligent driver model
LWR	Lighthill–Whitham–Richards
TFD	Triangular fundamental diagram
EXAT	Expressway Authority of Thailand
ITS	Intelligent transportation system
CTM	Cell transmission model
SHAP	SHapley Additive exPlanations
RF	Random forest
XGB/XGBoost	Extreme gradient boosting
RMSE	Root mean square error
MAE	Mean absolute error
R^2^	Coefficient of determination
PICP	Prediction interval coverage probability
MPIW	Mean prediction interval width

## Appendix A. Engineering Feature Models and Ensemble Formulas

This section formalizes the engineering of the inputs and the details of the ensemble set-up by combining traffic flow theory, microscopic behaviour models, and statistical/machine learning approaches. The raw inputs from the Intelligent Transportation System (ITS) detectors (speed, density, and flow) were transformed into theory-driven indicators that characterize congestion dynamics, while the ensemble models combined time-series backbones with tree-based learners for better predictive performance. The mathematical formulations for the engineered features, ensemble design, uncertainty quantification, and interpretability are described in the following section.

### Appendix A.1. Macroscopic Features and Short Derivation

To calculate the triangular fundamental diagram (TFD) and cell transmission model (CTM)/Lighthill–Whitham–Richards (LWR) for traffic flow, q(ρ) is modelled for each segment i adopted:

(A1) $$q\left(\rho \right)=min\{v_{f}\rho \;,\;w(\rho_{j}-\rho )\}$$

Free-flow speed is denoted by $v_{f}$, backward wave speed by ω>0, and jam density by $\rho_{j}$ [45,46,47,48]. Sending/receiving capacities with the discrete cells of length ∆x and step ∆t are determined as follows:

(A2) $$S_{i}\left(t\right)=min\{v_{f}\rho_{i}\left(t\right)\;,\;Q_{max}\}$$

(A3) $$R_{i+1}\left(t\right)=min\{\omega \left[\rho_{j}-\rho_{j+1}\left(t\right)\right]\;,\;Q_{max}\}$$

The maximum flow $Q_{max}$ is obtained at the critical density $\rho^{*}$, where the free-flow branch $\left(q=v_{f}\rho \right)$ and congested branch ($q=w(\rho_{j}-\rho$)) intersect. The traffic capacities hit their threshold before the onset of congestion. During the discretized update step, fluxes between adjacent cells are exchanged as the minimum sending capacity of upstream sites and the receiving capacity of downstream sites to ensure that the flow behaves consistently with demand and supply constraints, leading to

(A4) $$F_{i\to i+1}\left(t\right)=min\{\;S_{i}\left(t\right)\;,\;R_{i+1}\left(t\right)\}\;$$

Similarly to how Equations (A2) and (A3) are applied, $F_{i\to i+1}$ is substituted into conservation:

(A5) $$\rho_{i}\left(t+1\right)=\rho_{i}\left(t\right)-\frac{∆t}{∆x}F_{i\to i+1}\left(t\right)+\frac{∆t}{∆x}F_{i-1\to i}\left(t\right)$$

Equations (A2) and (A3) assign the maximum flux at every interface; Equation (A5) scaled density values as per the TFD (A1). The variables *sending_capacity*, *receiving_capacity*, *cell_outflow*, and *corrected_outflow* directly correspond to the fundamental quantities within the cell transmission model (CTM): S, R, and F. These quantities result from discretized traffic flow dynamics where upstream demand and downstream supply constrain inter-cell fluxes, and adjustments are made to consider phenomena such as capacity drop under congestion [47,48].

### Appendix A.2. Shockwave Speed and Three-Phase Kernel Surrogates

According to the kinematic wave theory, the shockwave between the upstream state ($\rho_{u}\;,\;q_{u}$) and downstream state ($\rho_{d}\;,\;q_{d})$ is expressed as follows:

(A6) $$w_{shock}(t)=\;\frac{q_{d}\left(t\right)-q_{u}(t)}{\rho_{d}\left(t\right)-\rho_{u}(t)}=\;\text{jam\_propagation\_speed}$$

The *jam_propagation_speed* feature is computed from adjacent cells or successive time pairs. The values of mean flow (*sensor_v2.mean_flow*) and mean density (*sensor_v2.mean_density*) were taken into consideration based on the shockwave formulation [45,46]. Based on Kerner’s three-phase theory, the following surrogate variables were derived: (i) free flow (F), (ii) synchronized flow (S), and (iii) wide-moving jams (W). Transition probabilities such as $P_{F\to S}(t)$ and $P_{S\to J}(t)$ were estimated from local thresholds in density (ρ), flow (q), and occupancy, as well as from the consistency of negative shockwave speed ${(w}_{shock}(t))$, representing upstream jam front propagation. The surrogate features directly map to the engineered variables *P_F_to_S* and *P_S_to_J* in [49].

### Appendix A.3. Microscopic Behavioural Features (Helbing)

Microscopic behavioural dynamics were simulated using the intelligent driver model (IDM) and a social force formulation. Inside the IDM framework, the desired inter-vehicle gap can be formulated as

(A7) $$S^{*}\left(v\;,\;∆v\right)=S_{0}+Tv+\frac{v∆v}{2\sqrt{a_{max}b}}$$

and the acceleration is updated as follows:

(A8) $$\dot{v}=\;a_{max}[1-{\left(\frac{v}{v_{0}}\right)}^{\delta }-({\frac{S^{*}\left(v\;,\;∆v\right)}{s})}^{2}]$$

where v denotes the vehicle speed, ∆v is the relative speed, and S represents the actual spacing. In this research, v was approximated from the *sensor_v2.mean_avg_speed* and ∆v from short-lag differences, which allowed behavioural surrogates to be estimated, such as *Repulsive_Force*, *Acceleration_To_Desired_Speed*, and *Total_Force* [50]. Complementing the IDM is the social force analogy, capturing interaction effects in which the net force acting on a vehicle is achieved as follows:

(A9) $$f_{i}=\frac{v_{i}^{0}e_{i}-V_{i}}{\tau }+\sum_{j\neq i}f_{ij}+\sum_{B}f_{iB}$$

where the first term represents relaxation with the aim of reaching a desired velocity, the second represents the pairwise interaction between vehicles, and the third is the boundary [51]. The scalar projections provided the numbered *Border_Influence* and *Total_Force* features, aggregated at the segment level, to describe microscopic driving responses relevant to congestion dynamics.

## Appendix B. Supplementary Statistical Analyses and Diagnostics

This appendix includes supplementary analyses, thus showing the robustness and interpretability of the proposed framework. The tables contain the results of the statistical significance tests and residual diagnostics; they are presented here to complement the main results of the study without disrupting the flow of the manuscript.

Statistical significance tests were performed while controlling for the residual temporal dependence, and the results are shown in Table A1. The bootstrap results show that the improvements in RMSE achieved by the hybrid ensemble forecasts over persistence and single-model baselines were rarely a matter of chance. By using block resampling instead of assuming independent errors, the analysis thus confirms that the gains from the ensemble are valid for the kinds of autocorrelation structures that traffic time series display.

**Table A1:** Results of paired moving-block bootstrap tests comparing ensemble models against baselines.

Comparison	RMSE (Test)	95% CI	*p*-Value	Block Length	w_xgb	α
Final Blend vs. persistence	0.099	[0.075, 0.125]	<0.001	2000	0.388	0.112
Final Blend vs. hybrid (constrained)	0.001	[−0.001, 0.003]	0.269	2000	0.388	0.112

Residual diagnostics were used to check for bias (Table A2), variance patterns, and dependence structures in the forecast errors. The measures of skewness and kurtosis suggest that the error distributions were near Gaussian, while the Durbin–Watson statistic suggests non-problematic autocorrelation. The low mean and variance of errors with high levels of these diagnostics testify to the production of stable forecasts by the Final Blend model across all regimes without systematic bias.

**Table A2:** Error summary for the Final Blend model on the test dataset, including distributional diagnostics.

N	Mean Error	Std Error	MAE	RMSE	Durbin–Watson	Skewness	Kurtosis Excess
2490	0.0020	0.1194	0.0671	0.1195	2.087	−1.167	8.294

The complementary nature of bootstrap significances (Table A1) and residual error diagnostics (Table A2) forms the foundation for more complete validation of the hybrid ensemble. Statistical testing provides assurance that the performance gains were unlikely to have a spurious origin, while distributional diagnostics assure that these improvements did not come at the expense of forecast stability or systematic forecast bias. Consequently, the ensemble’s superiority rests on a solid rigging of statistical inferences while also maintaining practical stability in the prediction errors.

The supplementary analyses offer complementary evidence that the accuracy improvements were statistically significant and robust. The bootstrap method substantiates otherwise true performance gains, not due to random chance under temporal dependence, and the residual diagnostics indicate an error distribution that is consistent, with no systematic bias ingrained within. These validations increase confidence in the reliability of the system and ensure that such stated improvements are characterized by rigorous statistical testing and transparent error analysis.
